# Goodness behind a wheel: moral judgment in realistic traffic simulations featuring Agents, Deeds, and Consequences

**DOI:** 10.3389/fpsyg.2026.1885843

**Published:** 2026-09-28

**Authors:** Dario Cecchini, Michael Pflanzer, Sam Cacace, Veljko Dubljevic

**Affiliations:** 1 Neurocomputational Ethics Research Group, North Carolina State University, Raleigh, NC, United States; 2 School of Psychology and Clinical Language Sciences, University of Reading, Reading, United Kingdom

**Keywords:** ADC model, autonomous vehicles ethics, moral psychology, traffic moral judgment, Virtual reality simulations

## Abstract

Investigating how different stakeholders evaluate traffic situations is essential for aligning autonomous vehicle (AV) decision-making with common-sense moral expectations. Yet, empirical studies on traffic moral judgment remain limited and almost exclusively focused on dramatic high-stakes situations. To address this gap, this paper presents two studies testing moral judgment in low-stakes ordinary traffic simulations. Drawing on the ADC model of moral judgment, both studies systematically manipulated key moral features: the character of the driver (Agent), adherence to traffic rules (Deed), and the presence or absence of a collision (Consequences). These components were embedded in a variety of stimuli, including textual vignettes, computer-generated video clips, and immersive audio-visual simulations. Data were collected from both expert ethicists (Study 1) and lay participants (Study 2). The results show that participants' moral judgments were consistently and strongly influenced by the character of the agent, whereas the effects of rule compliance and consequences were less robust. The mode of presentation (textual vs. audio-visual) significantly moderated these effects in mixed ways, suggesting that different formats may be better suited to conveying specific types of moral information. Notably, Study 1 revealed that expert ethicists tended to converge in their evaluations of low-stakes traffic scenarios, despite endorsing different moral theories. This convergence supports the feasibility of designing AV decision-making algorithms based on shared ethical judgments. Furthermore, the findings highlight that character-based considerations play a significant role in moral judgment of traffic scenarios—alongside deontological and consequentialist reasoning.

## 1 Introduction

The ethics of automated vehicles (AVs) have grown significantly in the last 10 years (Poszler et al., 2023). Key issues in this field include accountability and liability in the event of accidents, user privacy, cybersecurity, and fairness in decision-making. Among these, the problem of traffic decision-making has attracted particular attention from both the media and the public (Gill, 2021). This concern extends beyond rare, high-stakes dilemmas to encompass everyday decisions, such as how AVs should distribute risk among road users. Many scholars have argued that informing AV decision-making algorithms by common-sense moral judgments is essential for fostering public trust and ensuring the broad acceptability of this technology (Cecchini et al., 2024; Paulo and Kirchmair, 2025; Savulescu et al., 2021). This paper contributes to this aim.

To understand common-sense expectations with AVs, we need to investigate how stakeholders evaluate traffic situations. However, empirical research in this area remains limited (Poszler et al., 2023, p 16). To date, most studies have focused on moral judgments involving sacrificial dilemmas with AVs, framed through the influential “trolley problem” paradigm (Awad et al., 2018; Sütfeld et al., 2017; Faulhaber et al., 2019). On the other hand, everyday traffic situations—such as approaching intersections, changing lanes, or merging—have received far less scholarly attention. This lack of focus on routine, low-stakes scenarios represents a significant gap, given that such situations constitute many real-world driving contexts (Himmelreich, 2018; Borenstein et al., 2019; Cecchini et al., 2025). Arguably, low-stakes moral decisions precede and may lead to more drastic high-stakes situations, which makes this an important topic for research. Our studies address this gap by investigating how people evaluate the moral dimensions of ordinary traffic situations.

Several ethical frameworks have been proposed to implement moral decision-making into AVs (Geisslinger et al., 2023). Among them, the Agent-Deed-Consequence (ADC) model offers a promising integrative approach that draws from multiple moral theories and may align with common-sense intuitions (Cecchini et al., 2025; Cecchini and Dubljević, 2025; Dubljević, 2020). The ADC model is designed to accommodate both high- and low-stakes scenarios by synthesizing elements of virtue ethics, deontology, and consequentialism. While a recent empirical study has provided initial support for the model in the traffic domain (Pflanzer et al., 2025), further research is needed to assess its generalizability and robustness. The present article addresses this need by reporting two studies that test the model's core predictions using a larger and more diverse sample, and by presenting low-stakes traffic scenarios in three different formats: textual vignettes, computer-generated visual clips, and immersive audio-visual simulations.

Before illustrating and discussing our studies, we provide background on the issue of ethical decision-making in AVs, highlighting the main research gaps (Section 2). Then, in Section 3, we explore how virtual reality can enhance the ecological validity of empirical investigations into traffic-related moral judgment. Section 4 illustrates the ADC model of moral judgment as applied to traffic scenarios, followed by a presentation of our main hypotheses in Section 5. Section 6 presents our first study, while the second study is illustrated in Section 7. Finally, Section 8 summarizes the findings and discusses their implications and limitations.

## 2 Moral judgment in traffic

Driving automation involves automating some or all driving functions through hardware and software systems. High and full automation (SAE levels 4–5) refers to vehicles capable of performing all driving tasks autonomously (SAE On-Road Automated Driving Committee, 2021). These *autonomous* or *automated* vehicles, already operating in some U.S. cities (Waymo, 2024), raise important ethical concerns regarding decision-making in morally sensitive traffic situations. Accordingly, scholars have emphasized the need to embed ethical settings into AV systems to guide their behavior according to values such as safety, fairness, and passenger comfort (Lin, 2016; Millar, 2017; Geisslinger et al., 2023).

Ethical decision-making is increasingly regarded as a core design issue for AVs. Because AVs possess superior reaction times and data-processing capabilities compared to human drivers, they can potentially realize ethical goals more consistently and contribute to safer traffic environments. Moreover, embedding ethical settings into AV systems may foster public trust by ensuring that AVs are designed to minimize harm and treat road users with due care, especially given the negative impact that AV accidents can have on public perception of the technology.

Given the case for AV ethical settings, recent scholarship has focused on identifying the ethical factors that should guide AV behavior. To this end, some authors advocate a “hybrid” methodology (Cecchini et al., 2024; Paulo and Kirchmair, 2025; Savulescu et al., 2021) that combines laypeople's moral intuitions with expert judgment, including both intellectual experts (e.g., ethicists and policymakers) and practical experts (e.g., engineers and drivers). Aligning AV ethical settings with such a broad range of perspectives could help satisfy social expectations and promote trustworthy technology.

Aligning with this goal, several studies have investigated traffic moral judgment to identify common evaluative patterns among stakeholders. One of the most influential is the Moral Machine experiment (Awad et al., 2018), an online study that collected millions of responses worldwide by presenting unavoidable crash dilemmas in which participants had to choose between two outcomes depending on whether an AV swerved or stayed on course. The study revealed several recurring preferences, including saving humans over animals, more lives over fewer, and younger people over older ones.

Despite its influence, the Moral Machine paradigm has been criticized for relying on simplistic binary dilemmas and lacking ecological validity due to its highly gamified setting (Etienne, 2022; Cecchini et al., 2025). In response, recent research has increasingly shifted toward more ordinary and ecologically realistic traffic scenarios.

## 3 Toward more ecological validity: virtual reality and moral judgment

For many years, studies on moral judgment have relied on text-based descriptions of dilemmas to assess participants' responses. While this approach has produced important insights, it also has several limitations. First, textual scenarios lack contextual richness and situational cues that are often crucial for real-world moral judgment. Second, they may fail to elicit the emotional engagement—such as empathy or anger—that typically accompanies moral evaluation, limiting access to affective processes central to moral cognition. Third, textual descriptions are prone to interpretive variability due to differences in participants' background knowledge, language proficiency, and cultural norms. In addition, sacrificial dilemmas are especially sensitive to wording effects, which can significantly shape responses and reduce comparability across studies (McDonald et al., 2021).

Recent advances in virtual reality (VR) offer a way to address these limitations. VR can be defined as a computer-generated simulation that allows users to experience sensory stimuli in an interactive environment (Kardong-Edgren et al., 2019). VR systems vary in vividness (sensory richness) and interactivity (user engagement), ranging from desktop 3D videos to immersive head-mounted displays and virtual social interactions.

By providing immersive and naturalistic representations of morally salient situations, VR can improve the ecological validity of moral judgment research. Its vividness increases the likelihood that participants treat scenarios as real and engage the emotional processes involved in everyday moral decision-making (Patil et al., 2014). Unlike textual vignettes, VR preserves contextual detail and reduces interpretation bias and information loss in translation, thereby improving cross-cultural comparability.

Accordingly, a growing number of studies have used VR to investigate moral judgment in traffic contexts. Sütfeld et al. (2017), for example, examined driving decisions in a VR simulator in which participants navigated a two-lane road while encountering simultaneous obstacles—including objects, animals, and humans—and had to choose which to avoid. Consistent with the Moral Machine experiment, participants tended to prioritize sentient beings over objects, humans over animals, larger groups over individuals, and younger over older individuals. This broadly consequentialist pattern has been replicated in related VR studies (Faulhaber et al., 2019; Lucifora et al., 2020), although legal elements such as sidewalks and crosswalks can moderate willingness to sacrifice human life. Another study (Sütfeld et al., 2019) compared moral judgments across textual vignettes, desktop video, and immersive VR, finding no significant differences across modalities in sacrificial decisions.

Although these studies represent an important advance in ecological validity relative to earlier work such as the Moral Machine experiment, they focus primarily on high-stakes sacrificial dilemmas involving life-and-death trade-offs. By contrast, everyday low-stakes traffic decisions—such as lane changes, merging, or approaching intersections—remain understudied.

The present studies address this gap by using VR-generated video clips to examine low-stakes (non-life-threatening) traffic situations. Participants were exposed to simulations in two formats: low-immersion desktop video (Study 1) and a more immersive 360-degree environment (Study 2). Comparing judgments across levels of immersion, and against traditional textual vignettes, allows us to assess how presentation mode influences moral evaluation.

## 4 The ADC model of traffic moral judgment

To study low-stakes traffic scenarios, we require an experimental paradigm that moves beyond the traditional focus on life-and-death trade-offs between principles and consequences, incorporating insights from person-centered approaches (Uhlmann et al., 2015). The ADC model of traffic moral judgment provides such a framework.

The core idea of the ADC model is that moral judgment arises from the evaluation of three components: the character of the actor involved (Agent, A), the nature of the action (Deed, D), and the outcome of the situation (Consequence, C) (Dubljević and Racine, 2014). Overall moral evaluation depends on the valence of each component: positive valence typically yields more favorable judgments, while negative valence leads to less favorable evaluations.

A key theoretical strength of the ADC model is its integration of competing ethical traditions. Virtue ethics emphasizes the agent's character, deontology focuses on the intrinsic nature of actions and rule compliance, and consequentialism prioritizes outcomes. These perspectives assign different weights to A, D, and C, which can lead to divergent judgments in cases of conflict.

This tripartite structure is especially useful for analyzing moral tensions in traffic contexts, where driving style, rule compliance, and risk distribution often conflict. In addition, decomposing moral judgment into discrete components has practical advantages for computational modeling of artificial agents, as it enables complex moral reasoning to be represented in a structured and quantifiable way (Pflanzer et al., 2023, p. 924). The ADC framework is also highly adaptable to experimental research, since A, D, and C can be systematically manipulated in textual or audiovisual stimuli, facilitating large-scale empirical studies (Cecchini et al., 2025).

Recent work has applied the ADC model to traffic moral judgment (see Figure 1 for an overview). In this domain, the Agent component refers to the intentions and character traits of the drivers involved. For example, observers may evaluate a driver positively when rushing an injured passenger to the hospital (A+), and negatively when a vehicle is speeding under the influence of alcohol (A–). Although AVs are not moral agents in the human sense, they exhibit functional analogs of intention and character through their goal structures and driving styles (e.g., acceleration, braking, and interaction patterns). These features can influence perceptions of moral character and shape judgments of AV behavior.

**Figure 1:**
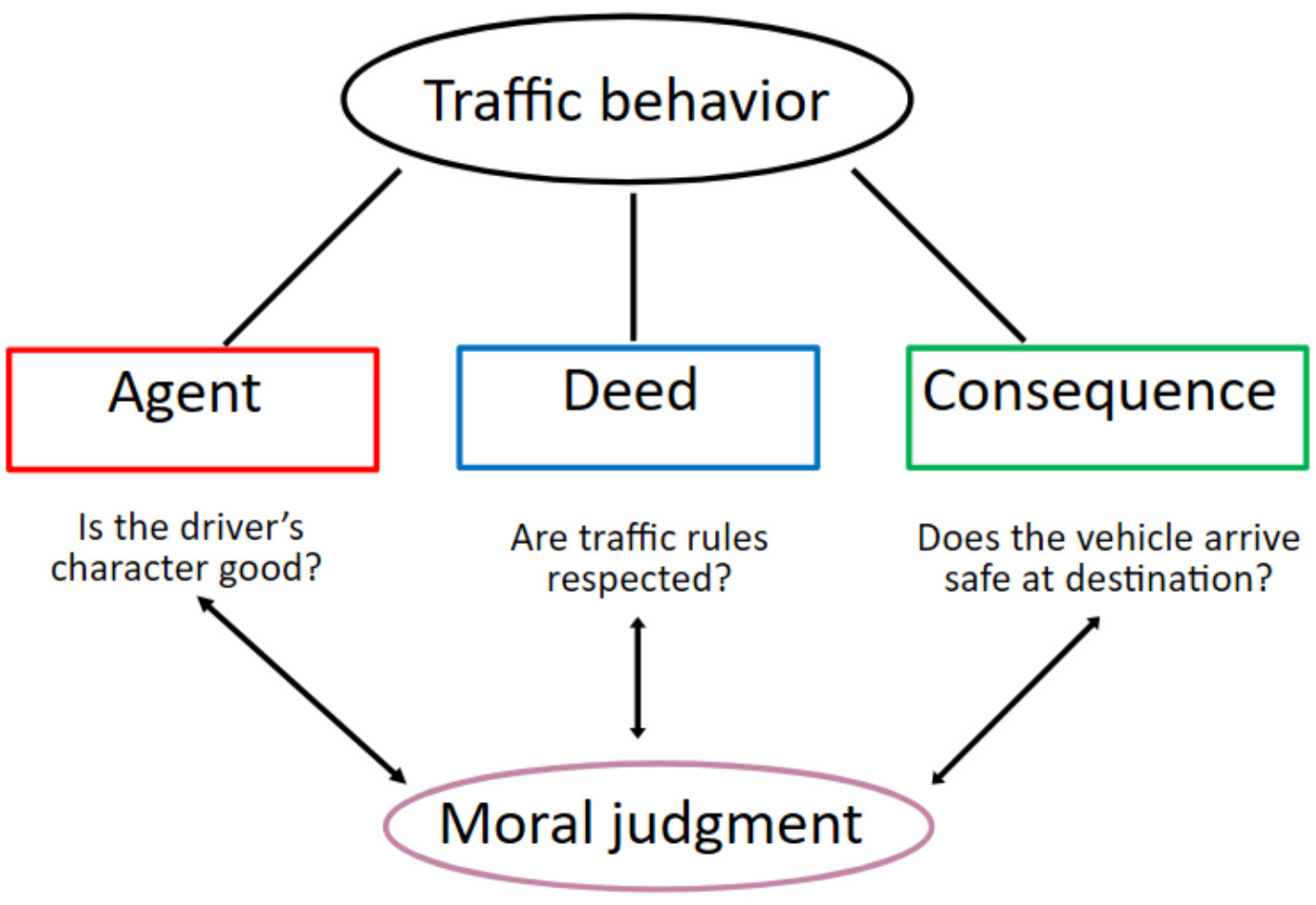
The ADC model of traffic moral judgment.

The Deed component concerns whether actions comply with moral or legal norms, such as speed limits, traffic signals, and right-of-way rules. For AVs, rule adherence constitutes good deeds (D+), while violations constitute bad deeds (D–). While rule compliance is crucial for safety and predictability, public acceptance of AVs also depends on their ability to balance rule-following with contextual flexibility, as human drivers sometimes do in exceptional circumstances.

The Consequence component captures the outcome of actions, such as safe arrival (C+) or accidents (C–). Since a central aim of AV development is reducing traffic accidents, consequences play a major role in moral evaluation and in shaping public expectations about AV behavior.

The ADC model has received growing empirical support. Studies outside the traffic domain show that people reliably distinguish between agent, deed, and consequence when making moral judgments (Dubljević et al., 2018). More recently, Pflanzer et al. (2025) found support for the ADC model's core hypotheses in the traffic domain using textual vignettes and a sample of professional philosophers. However, it remains unclear whether these findings generalize across different stakeholder groups and modes of presentation.

The present studies address these limitations in two ways: first, by extending the sample beyond professional philosophers to include non-expert participants; and second, by increasing ecological validity using immersive VR traffic simulations instead of purely textual vignettes.

## 5 The present studies: hypotheses and research questions

The primary goal of the present studies is to examine whether people's moral expectations in ordinary traffic situations align with the core hypotheses of the ADC model. In doing so, we aim to advance our understanding of moral judgment in traffic contexts, which constitutes a crucial first step toward the ethical programming of AV decision-making.

We predict consistent main effects of Agent, Deed, and Consequence components on moral judgment, regardless of the sample and presentation format. More precisely:

- **H1:** positively portrayed drivers (A+) will have higher moral acceptability as compared to negatively portrayed drivers (A–).
- **H2:** traffic actions respecting rules (D+) will have higher moral acceptability as compared to actions violating rules (D–).
- **H3:** traffic actions resulting in no collision (C+) will have higher moral acceptability as compared to traffic actions resulting in a collision (C–).

A secondary aim of the present studies is to examine whether individual moral preferences—namely, for virtue ethics, deontology, or consequentialism—moderate the influence of the Agent, Deed, and Consequence components on moral acceptability judgments. Prior research has yielded mixed findings. Some studies applying the ADC model outside the traffic domain found significant interaction effects between moral components and individuals' ethical orientations (Dubljević et al., 2018). In contrast, the only study to date that has applied the model to low-stakes traffic scenarios found no such moderating effect (Pflanzer, Cecchini et al., 2025). Further investigation is needed to assess whether different ethical standpoints converge toward common guidelines for AVs. Therefore, we investigate the following questions:

- **R1:** will preference for *virtue ethics* moderate the effect of drivers' character (A) on moral acceptability judgments?
- **R2:** will preference for *deontological ethics* moderate the effect of rule violations (D) on moral acceptability judgments?
- **R3:** will preference for *consequentialism* moderate the effect of traffic collision (C) on moral acceptability judgments?

In addition to examining how individuals' moral preferences shape traffic-related moral judgments, the present studies also investigate how the mode of presentation of scenarios influences moral evaluation. Because real-world traffic decisions are typically assessed visually by external observers, this factor is essential for understanding moral expectations. Accordingly, we test whether presenting the same moral scenario either textually (i.e., as a written description) or visually (i.e., as a VR-generated clip) moderates the influence of the three moral components.

Previous research has yielded mixed findings: while some studies using high-stakes trolley dilemmas suggest that visual and immersive formats tend to promote more consequentialist judgments (Patil et al., 2014), others—particularly those involving high-stakes traffic scenarios—have found no significant effect of presentation mode (Sütfeld et al., 2019). To the best of our knowledge, no study thus far has investigated the effect of the presentation mode on the moral perception of a driver's character. Thus, we address the following questions:

- **R4:** will the presentation mode (Textual vs. A/V) moderate the effect of drivers' character (A) on moral judgment?
- **R5:** will the presentation mode (Textual vs. A/V) moderate the effect of traffic violation (D) on moral judgment?
- **R6:** will the presentation mode (Textual vs. A/V) moderate the effect of traffic collision (C) on moral judgment?

## 6 Study 1: moral judgment of professional ethicists

Our first experiment tested the core hypotheses of the ADC model (H1–H3) in the context of low-stakes traffic scenarios. For this study, we recruited a large sample of professional ethicists, i.e., scholars who have actively contributed to a notable academic journal in bioethics. We selected this sample for two main reasons. First, as experts in moral reasoning, ethicists provide a valuable perspective for informing the ethical design of emerging technologies such as AVs, in line with the previously mentioned hybrid methodology. Second, we expected that participants with formal ethical training would hold consciously endorsed and relatively well-defined preferences for moral theories. This makes them an ideal population for examining whether differing educated intuitions—virtue-oriented, deontological, and consequentialist—lead to convergent or divergent moral judgments in applied scenarios (R1–R3).

To isolate the influence of the mode of presentation on moral judgments (R4-R6), we divided the sample into two groups: one assessing traditional textual vignettes, the other judging desktop video clips of the same scenarios.

### 6.1 Methods

#### 6.1.1 Participants

Following previous studies (Pierson et al., 2024), we contacted nearly 4,000 professional ethicists using a mailing list of scholars who had submitted work to a prominent academic journal in bioethics, inviting them to participate in our online surveys hosted on Qualtrics. With this number of solicitations, we hoped for at least a 20% response rate, corresponding with 400 participants for each condition. In line with our IRB approval (REDACTED), all participants completed an informed consent form. Approximately half of the participants received a survey containing traditional textual vignettes (Textual Group), while the other half received a survey with audio-visual video clips (A/V Group).

We recruited 796 participants in total: 386 completed the survey with A/V vignettes, and 410 completed the version with textual vignettes. From this initial sample, 208 participants (26.1%) were excluded for completing less than 67% of one or more measures, and 96 (12.1%) were excluded for showing linear response patterns (less than 0.5 SD variability across responses).^1^ An additional 35 participants (4.4%) were flagged by Qualtrics' ReCAPTCHA bot-detection system. The final sample consisted of 457 participants (43.1% male, 54.5% female, 2.4% non-binary), with 226 in the A/V condition and 231 in the textual condition.

#### 6.1.2 Design, procedures, and materials

This online quasi-experiment employed a 2 (Presentation mode: Textual vs. A/V) x 2 (Agent: Positive vs. Negative) x 2 (Deed: Positive vs. Negative) x 2 (Consequences: Positive vs. Negative) between-subjects design. Each participant was randomly assigned to one of the resulting combinations of conditions.

After responding to some demographic questions and the attention check, participants completed a modified 11-item version of the Preference for Precepts Implied in Moral Theories (PPIMT) scale, designed to assess individual moral preferences (Dubljević et al., 2022). The scale comprises 11 items related to the three major frameworks in moral philosophy: Virtue Ethics (4 items), Deontology (4 items), and Consequentialism (3 items). All PPIMT measures begin with the phrase, “When thinking about what is moral or immoral in a situation, it is important to me whether the involved persons….” Participants were then asked to rate their agreement on a range from “disagree very much” [1] to “agree very much” [7], with the remaining clauses that pertain to Agents (Virtue Ethics), Deeds (Deontology), and Consequences (Consequentialism).

To account for potential anomalies in moral judgment, we assessed participants' psychopathic traits using the 26-item Levenson Self-Report Psychopathy Scale (LSRP) (Falkenbach et al., 2007; Gummelt et al., 2012). The LSRP includes two subscales: primary psychopathy (16 items), which measures traits such as callousness, manipulativeness, and lack of empathy; and secondary psychopathy (10 items), which captures impulsivity, poor behavioral control, and social deviance. Participants responded using a 4-point Likert scale ranging from 1 (“Strongly Disagree”) to 4 (“Strongly Agree”). Following the standard LSRP scoring procedure, 11 items were reverse-coded (e.g., “Even if I were trying hard to sell something, I wouldn't lie about it”) to ensure that higher scores consistently reflected higher levels of psychopathic traits. The total psychopathy score was calculated by summing responses across all 26 items, resulting in a possible range from 26 to 104.

Following these questions, participants were presented with moral vignettes. We used two low-stakes traffic scenarios slightly adapted from a prior study (Pflanzer et al., 2025): one involving a mother driving her child to school (Intersection scenario) and the other featuring a man transporting a cat to the veterinary hospital (Pet scenario) (see Appendix for the full textual version of the vignettes). Each scenario consisted of eight vignettes representing all combinations of moral component valence (A+/–, D+/–, C+/–), in line with the ADC model.

In each vignette, the protagonist is described as having either a virtuous or vicious character (A+/–), obeying or violating a traffic rule (D+/–), and producing either positive or negative low-stakes consequences (C+/–). For example, in the Intersection scenario, the protagonist is either a caring (A+) or abusive (A–) parent rushing to get their child to school. They encounter a yellow traffic light and either obey the law by stopping (D+) or speed up to beat the red light (D–). As a result, the child either arrives on time (C+) or the car is involved in a minor incident, causing the child to miss school (C–).

Each participant was randomly assigned two vignettes—one from the Pet scenario and one from the Intersection scenario. To minimize contrast, anchoring, and order effects, the sequence in which the vignettes were presented was randomized. Depending on the assigned condition, participants received either textual or A/V versions of the vignettes. In the A/V condition, desktop video clips were embedded directly into the survey, and participants were instructed to watch each clip before responding.

A/V vignettes were fully based on the textual vignettes and were developed using the Unity Real-Time Development Platform (version 2020.3.18f1) (Figure 2). The A/V stimuli—including environments, characters, dialogues, and animations—were sourced from public domain files available on the Unity Asset Store and Mixamo.com or were custom-built by our laboratory developers using Unity and Blender.

**Figure 2:**
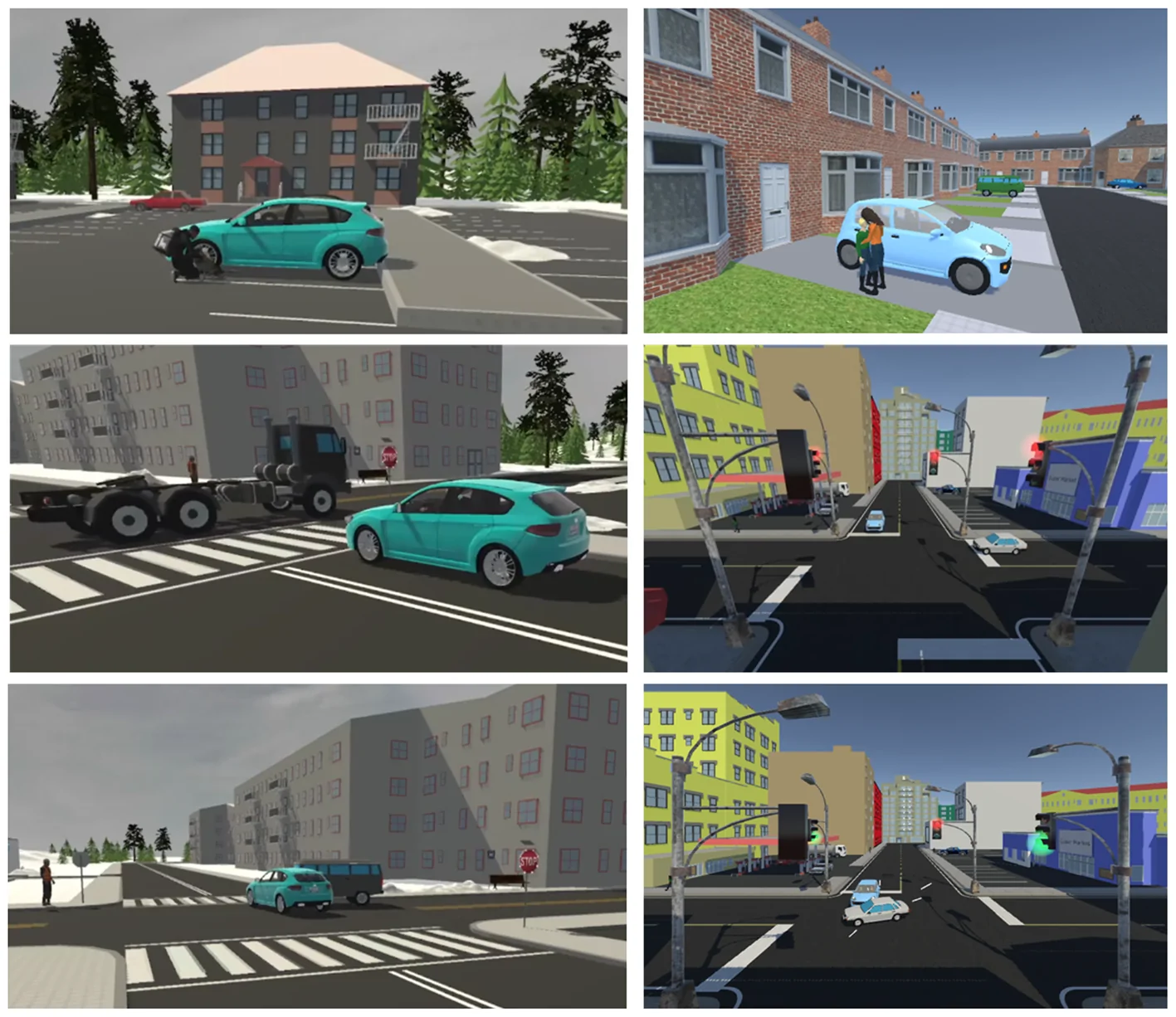
Audio-visual clips of the Pet (on the left) and Intersection (on the right) scenarios, featuring the driver's character (first line), traffic rule compliance or violation (second line), and collisions (third line).

After reading or watching each vignette, participants were asked to consider all circumstances of the narrative and to rate both the *personal* and *social* moral acceptability of what the agent did in the situation (e.g., “for *you personally* [*society*], how morally acceptable is what the man did in the situation?”). Response options varied from “not at all acceptable” [1] to “completely acceptable” [10]. We asked for both social and personal ratings because people sometimes have different expectations for each value. To assess whether the two measures converged, we conducted a Pearson's *r* correlation test, which suggested that both measures reflect a single underlying latent construct (*r* > 0.88, *p* < 0.001). Accordingly, we averaged the two into a composite moral judgment score.

#### 6.1.3 Analytic plan

Primary statistical analyses were conducted in SAS 9.4 (SAS Institute Inc. Cary, NC, USA). Mixed-effects models were estimated using PROC MIXED to account for repeated observations within participants, with moral judgment ratings entered as dependent variables and the relevant vignette characteristics, presentation conditions, and participant-level factors entered as fixed effects according to each analysis. General linear models were estimated using PROC GLM where appropriate. Statistical significance was evaluated at α = 0.05, and estimated effects are reported with standard errors, effect sizes, and exact *p* values where applicable.

To test H1–H3 and R4–R6, mixed-effects models examined the effects of Agent (A), Deed (D), Consequence (C), and presentation mode (Textual vs. A/V), including their relevant interactions. Least-squares means and pairwise comparisons were used to estimate component-valence effects overall and within presentation modes. Where applicable, denominator degrees of freedom were estimated using Kenward-Roger approximation.

To examine whether individual moral preferences moderate the influence of ADC components on moral judgment (R1–R3), we estimated separate mixed-effects models testing the interaction between participants' moral framework and each corresponding ADC component. Planned contrasts compared positive and negative component valences within their theoretically corresponding frameworks: Agents within virtue ethics, Deed within deontology, and Consequence within consequentialism. Exploratory analyses additionally examined moderation using participants' continuous PPIMT factor scores. For framework-specific analyses, participants were classified according to their highest standardized PPIMT factor score when it exceeded the next-highest score by at least 0.3—a threshold reflecting a moderate effect size (Cohen, 1988). Participants not meeting this criterion were classified as Pluralist and excluded from framework-specific tests (but retained for all other analyses).

Exploratory analyses additionally examined whether LSRP psychopathy scores or psychopathy classification were associated with moral judgment or moral-framework assignment.

Supplementary analyses were conducted in R version 4.5.2 (Vienna, Austria; https://www.R-project.org/). Using R (*pwr* package) (Champely, 2020), we conducted a sensitivity power analysis to determine the smallest detectable effect sizes given the final analyzed sample (*n* = 457). Assuming α = 0.05 and 80% power, the design could detect effects of approximately *d* = 0.19 (A, D, C factors; *f* = 0.10, η^2^_*p*_ = 0.01) and a between-subjects Group (A/V vs. Textual) effect of *d* = 0.26 (*f* = 0.13, η^2^_*p*_ = 0.02). These values correspond to small effect sizes under Cohen's conventions, indicating sensitivity to effects of relatively modest magnitude.

For focal non-significant findings, supplementary Bayesian model comparisons were conducted in R using the *BayesFactor* package (version 0.9.12-4.8) (Morey and Rouder, 2026). Bayesian mixed-model comparisons were estimated using lmBF() with the package's default JZS prior specification and participant treated as a random factor. For each focal non-significant effect, a model containing the effect of interest was compared with an otherwise matched reduced model excluding that effect. Bayes factors are reported as BF_01_ and BF_10_, where BF_01_ represents the relative evidence for the reduced model over the model containing the focal effect, and BF_10_ represents the reciprocal comparison. These Bayesian analyses were used to distinguish evidence favoring a null effect from non-significant results for which the available evidence remained inconclusive.

### 6.2 Results

#### 6.2.1 Main effects of Agent, Deed, and Consequences (H1–H3)

The mixed-effects analysis revealed significant main effects of Agent (A) and Consequences (C), but not on Deed (D) (Table 1). Participants judged positively characterized agents substantially more favorably than negatively characterized agents (Δ*M* = 2.39, *SE* = 0.14, *p* < 0.001, *d* = 1.21), providing strong support for H1. Positive consequences were likewise judged more favorably than negative consequences (Δ*M* = 1.05, *SE* = 0.14, *p* < 0.001, *d* = 0.53), supporting H3. By contrast, the overall difference between positive and negative deeds was small and non-significant (Δ*M* = 0.14, *SE* = 0.11, *p* = 0.325, *d* = 0.07) and therefore did not support H2.

**Table 1:** For Deed, the overall effect was not significant, but simple effects within each presentation mode were significant in opposite directions (Textual: Δ*M* = −1.37, *SE*=0.20; AV: Δ*M* = 1.66, *SE*=0.20).

Main effects	*M*_positive_	*M*_negative_	Δ*M*	*SE*	*F*	df	*p*	Cohen's *d*
Agent	5.49	3.10	2.39	0.14	274.2	853	<0.001	1.21
*Textual*	5.01	3.95	1.06	0.20	27.0	223	<0.001	0.50
*A/V*	5.97	2.25	3.72	0.20	334.5	218	<0.001	1.88
Deed	4.37	4.23	0.14	0.11	0.97	852	0.325	0.07
*Textual*	3.79	5.17	−1.37	0.20	45.7	227	<0.001	0.69
*A/V*	4.94	3.28	1.66	0.20	65.6	227	<0.001	0.84
Consequence	4.82	3.77	1.05	0.14	53.4	840	<0.001	0.53
*Textual*	5.47	3.50	1.97	0.20	94.5	235	<0.001	0.99
*A/V*	4.18	4.05	0.13	0.20	0.42	235	0.517	0.07
**Interaction effects**	Δ*M*_textual_	Δ*M*_A/V_	ΔΔ***M***	***SE***	***F***	**df**	***p***	
A x Presentation	1.06	3.72	2.66	0.29	84.9	853	<0.001	0.09
D x Presentation	−1.37	1.66	3.03	0.29	110.1	852	<0.001	0.11
C x Presentation	1.97	0.13	1.84	0.29	40.8	840	<0.001	0.05

Cohen's *d* was calculated as Δ*M* / √(residual variance). Partial η^2^ values were derived from the *F* statistic using η^2^_*p*_ = *F* / (*F* + df_2_). All means are model-estimated least-squares means (LSMs).

A supplementary Bayesian model comparison further favored the model without a marginal Deed-valence, BF01 = 12.57 (BF10 = 0.080), indicating substantially greater evidence for the model excluding the overall Deed effect than for the model including it.

Given these mixed findings, we re-examined H1–H3 separately within each presentation mode (see Figure 3 and Table 1 for a summary). These follow-up analyses revealed that an unexpected *negative* difference in moral judgment scores between positive (3.79) and negative (5.17) deeds for the textual presentations (Δ*M* = −1.37, *SE* = 0.20, *p* < 0.001) canceled out the *positive* difference in moral judgment scores between positive (4.94) and negative (3.28) moral judgment scores for the A/V presentation (Δ*M* = 1.66, *SE* = 0.20, *p* < 0.001), thereby resulting in a non-significant difference for the overall sample. Additionally, it revealed that the difference between positive (4.18) consequences and negative (4.05) consequences in the A/V presentation mode was not significant (Δ*M* = 0.13, *p* = 0.517). A supplementary Bayesian mixed-model comparison favored the model without a Consequence-valence effect within the A/V condition, BF01 = 6.87 (BF10 = 0.146).

**Figure 3:**
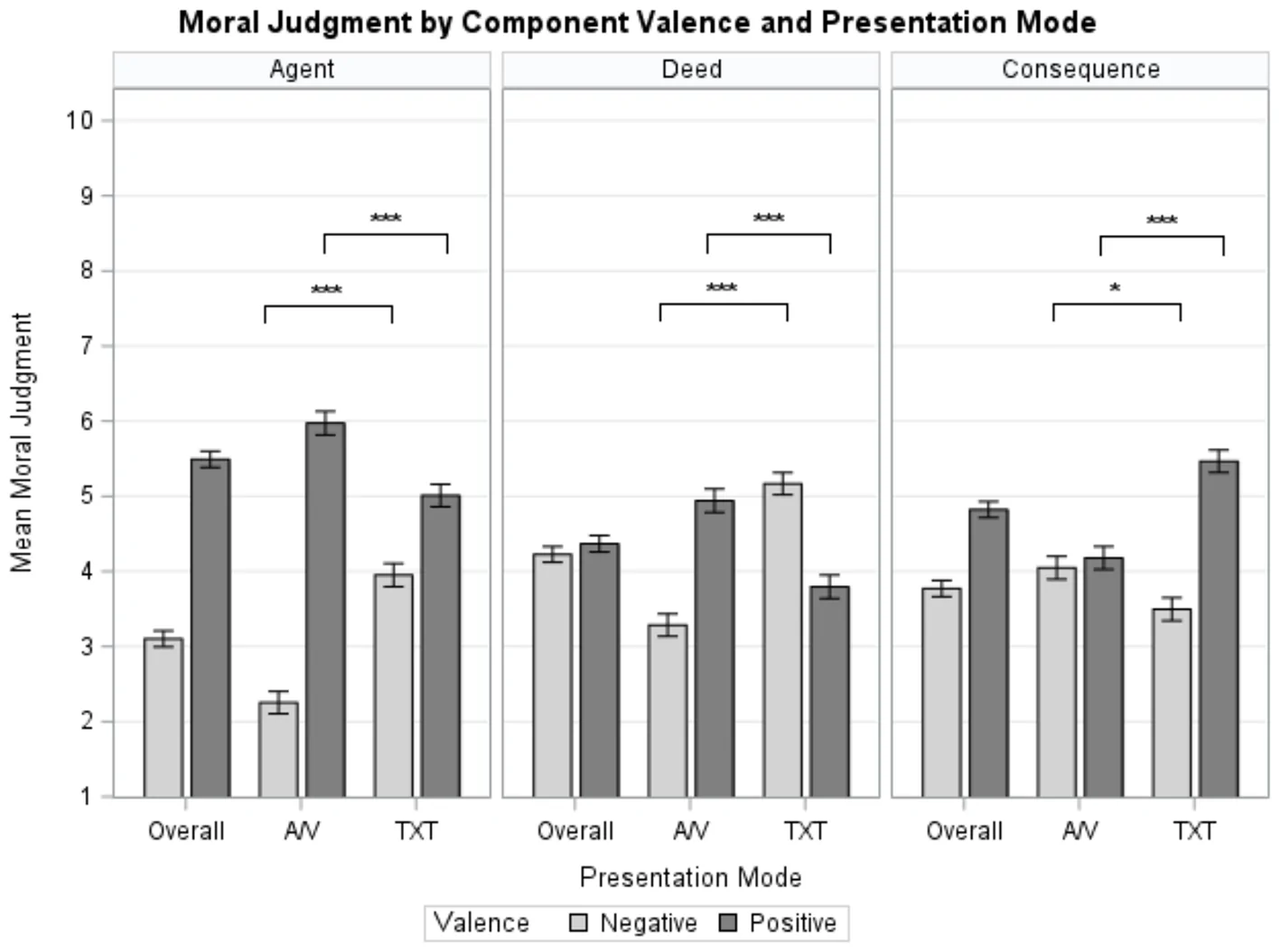
Model-estimated mean moral judgment scores by ADC component valence and presentation mode, including marginal means. Error bars represent ±1 standard error. Brackets and asterisks indicate significant differences between A/V and TXT presentation modes within the corresponding valence condition. *p* < 0.05; *p* < 0.001.

#### 6.2.2 Moderation of Agent, Deed, and Consequences by individual differences (R1–R3)

A confirmatory factor analysis of the PPIMT indicated limited fit for the theorized three-factor structure, χ^2^(44) = 322.10, *p* < 0.001; RMSEA = 0.12, 90% CI (0.11, 0.13). Standardized factor loadings were moderate to strong across all items (Table 2), and internal consistency was high for all three subscales (ω = 0.846 for Virtue Ethics, ω = 0.846 for Deontology, and ω = 0.832 for Consequentialism).

**Table 2:** PPIMT factor loadings using confirmatory factor analysis.

Variable	Factor	Std. loading (λ)	*SE*	*p*
MP1	F1	0.644	0.03	<0.001
MP11	F1	0.830	0.02	<0.001
MP12	F1	0.921	0.02	<0.001
MP15	F1	0.623	0.03	<0.001
MP5	F2	0.750	0.03	<0.001
MP7	F2	0.797	0.02	<0.001
MP10	F2	0.675	0.03	<0.001
MP13	F2	0.817	0.02	<0.001
MP6	F3	0.856	0.02	<0.001
MP8	F3	0.751	0.03	<0.001
MP14	F3	0.757	0.03	<0.001

To ensure robust classification of participants' moral orientation, we required that the highest standardized factor score exceed the next highest by at least 0.3. Applying this conservative criterion, 437 participants (95.6%) were assigned to one of three Frameworks: 149 (32.6%) to Deontology, 146 (32.0%) to Virtue Ethics, and 142 (31.1%) to Consequentialism. The remaining 20 participants (4.4%) did not meet the threshold and were labeled “Pluralist.” These participants were included in overall analyses but excluded from framework-specific hypothesis testing.

None of the Component × Framework interactions reached statistical significance. Exploratory analyses using participants' continuous PPIMT factor scores (Moral Judgment = A × A_score + D × D_score + C × C_score). None of the Component x Framework interactions reached statistical significance.

Exploratory analyses yielded no significant associations between LSRP scores or psychopathy classification and either moral judgment or moral-framework assignment.

#### 6.2.3 Moderation of Agent, Deed, and Consequences by presentation mode (R4–R6)

Presentation mode significantly moderated all three ADC components (Table 1; Figure 3). The Agent x Presentation interaction was significant, *F* (1, 853) = 84.9, *p* < 0.001. The difference in moral judgment between A+ and A– was significantly greater in the A/V condition (Δ*M* = 3.72, *SE* = 0.20) than in the Textual condition (Δ*M* = 1.06, *SE* = 0.20) (Figure 3, Table 1), supporting R4.

The Deed x Presentation interaction was also significant, *F* (1, 852) = 110.1, *p* < 0.001. Moreover, the effect reversed directions across presentation modes: A/V participants judged D+ more favorably than D- (Δ*M* = 1.66, *SE* = 0.20), whereas Textual participants judged D+ less favorably than D– (Δ*M* = −1.37, *SE* = 0.20). This interaction supports R5 while revealing an unexpected reversal in the Textual condition.

Finally, the Consequence x Presentation interaction was significant, *F* (1, 840) = 40.8, *p* < 0.001. The C+ vs. C– was significantly greater in the Textual condition (Δ*M* = 1.97, *SE* = 0.20) than in the A/V condition (Δ*M* = 0.14, *SE* = 0.20). Thus, presentation mode significantly moderated the Consequence effect, supporting R6, although the Consequence effect within the A/V condition was not distinguishable from zero.

### 6.3 Study 1 discussion

In this study, involving a large sample of professional ethicists, we found mixed evidence for the core principles of the ADC model of traffic moral judgment. Overall, participants evaluated scenarios involving virtuous drivers more positively than those involving vicious drivers, indicating a robust influence of agent character across both textual and visual presentations. By contrast, effects related to outcomes were weaker and depended on the mode of presentation: scenarios without collisions were evaluated slightly more positively overall, but this effect was primarily driven by the textual condition and was not evident in the A/V condition. The influence of rule compliance was also inconsistent. While audiovisual scenarios showed a tendency to favor rule-following behavior over violations, textual scenarios unexpectedly produced the opposite pattern, with rule violations sometimes receiving more positive evaluations. This divergence suggests that the impact of traffic rules on moral judgment is highly sensitive to presentation format and may even reverse depending on how the scenario is conveyed, in contrast with prior findings using similar materials and samples (Pflanzer et al., 2025).

Several interpretations may account for this pattern. First, textual descriptions may have reduced the salience of rule-violating behavior relative to other narrative elements, particularly the character of the agent and the consequences of the action. Second, participants may have interpreted some of the rule violations as minor or contextually justified infractions rather than clear moral transgressions. Finally, the textual format may have introduced greater variability in how participants interpreted the scenarios, allowing some readers to mentally reconstruct circumstances that made the violation appear reasonable. Because these interpretations cannot be distinguished within the present design, future research should examine this effect using more tightly controlled manipulations of rule violations and additional measures assessing participants' perceptions of rule legitimacy.

The analysis of H1–H3 also sheds light on the role of vignette presentation mode in moral judgment, addressing research questions R4–R6. We found that presentation format (A/V vs. textual) differentially highlights specific types of moral information. In particular, A/V presentations appear to enhance the salience of agent character and rule compliance, while textual descriptions more strongly emphasize the moral relevance of outcomes.

Finally, we tested whether individual preferences for moral theories moderated moral judgment across the three components (R1–R3). Consistent with prior findings (Pflanzer et al., 2025), we found no interaction between moral orientations (virtue ethics, deontology, and consequentialism) and moral evaluations of traffic scenarios. This suggests a convergence in judgments among professional ethicists, regardless of their theoretical commitments. One possible explanation is the low-stakes nature of the scenarios, which involve ordinary traffic situations rather than high-conflict sacrificial dilemmas between competing moral principles.

Effect sizes for main and interaction effects were generally in the small-to-moderate range. This may be due, in part, to the limited number of observations per participant. Because each participant evaluated only one vignette per scenario, there were no within-subject comparisons of component valence, which likely increased residual variance and constrained the model's sensitivity to fixed effects. Our next study addresses this limitation by using a within-subject design.

## 7 Study 2: moral judgment in immersive traffic simulations

Our second study significantly differs from Study 1 in terms of sample characteristics, use of VR, and experimental design. In this experiment, we recruited college students with no formal background in moral philosophy, providing a contrast to the expert sample of professional ethicists. Furthermore, we increased the ecological validity of the scenarios by presenting low-stakes traffic situations in a more immersive format, projecting video clips on a 360-degree screen. Unlike Study 1, this study employed a within-subject design, allowing participants to observe multiple variations of the Agent, Deed, and Consequence components across different A/V stimuli.

### 7.1 Methods

#### 7.1.1 Participants

Participants of this study were undergraduate and graduate students from a large public university in REDACTED. A total of 206 students initially took part in the study. Classes were alternately assigned to Group A or Group B, each of which viewed a different half of the vignettes. All participants received course credit or extra credit for their participation. We did not conduct power analyses to calculate our projected sample size; our total sample instead reflects enrolled students, eligible and willing to participate.

Like Study 1, we excluded participants with less than 67% completion across one or more surveys (*n* = 34) less than 10 of 15 PPIMT prompts or less than 30 of 45 Vignette prompts. 26 participants providing linear response patterns (those with less than 0.5 standard deviation for either the PPIMT or the Vignette responses) were also excluded to remove potentially careless responses (Leiner, 2019). At the end, 146 participants (48% male, 52% female) were retained for analysis (Group A: *n* = 73; Group B: *n* = 73).

#### 7.1.2 Design, procedure, and materials

For this study, we employed a 2 (Agent: Positive vs. Negative) x 2 (Deed: Positive vs. Negative) x 2 (Consequences: Positive vs. Negative) within-subjects design, in which each subject assessed multiple combinations of experimental conditions.

The experiment was conducted in an immersive visual and auditory gallery equipped with eight high-definition projectors and a specially configured computer (Figure 4). This setup enabled the presentation of 360-degree audio-visual content, providing participants with a fully immersive experience. Groups of up to 25 participants entered the room at a time and were instructed to individually complete an electronic survey (hosted on Qualtrics) on a tablet. In accordance with the approved IRB protocol (REDACTED), participants provided written informed consent and were informed that the content could be emotionally impactful and were reminded of their right to leave the room at any time should they feel discomfort.

**Figure 4:**
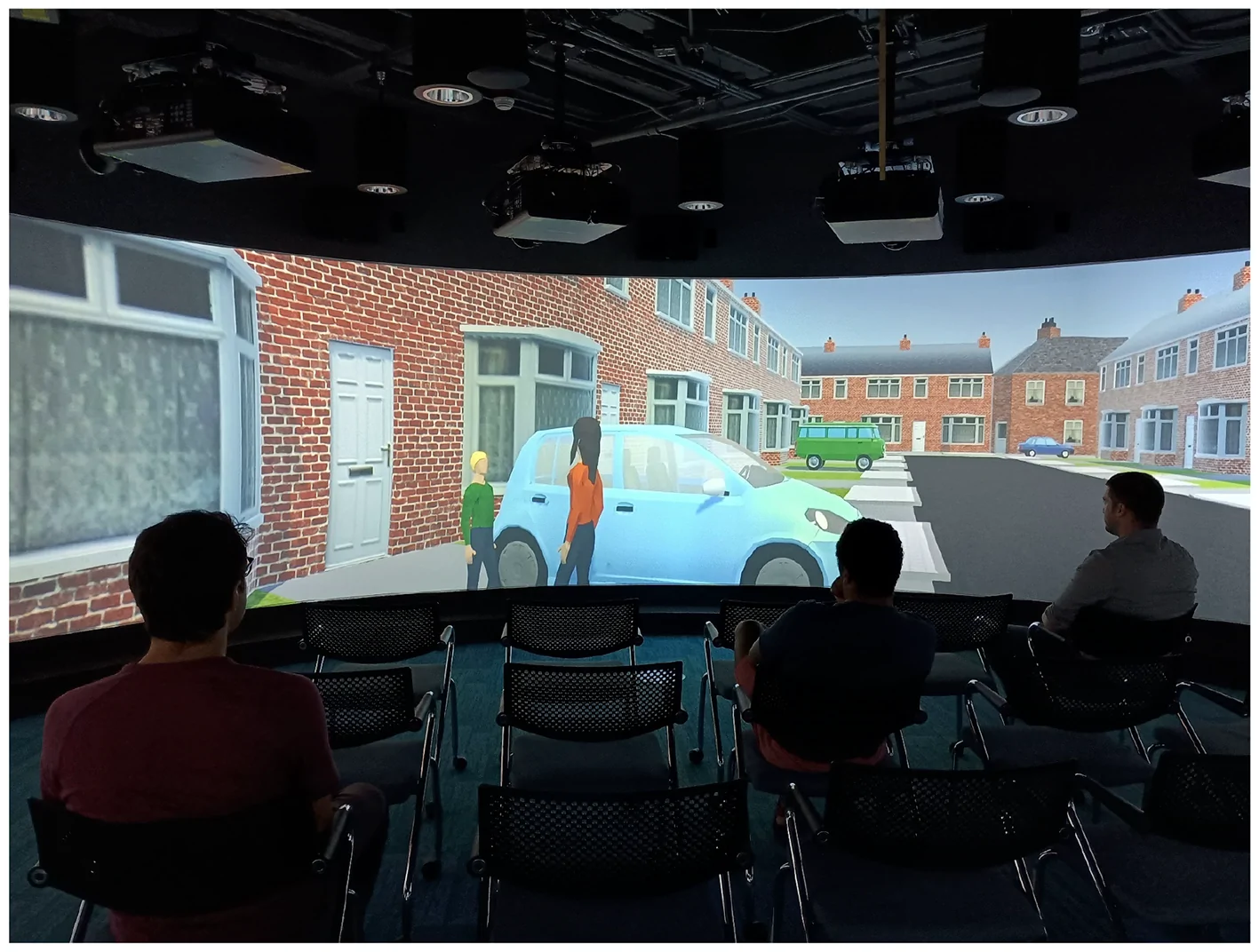
The immersive visualization gallery employed for Study 2.

Before viewing the simulations, subjects provided demographic information and completed the PPIMT scale to assess their individual moral preferences. Unlike in Study 1, no assessment of psychopathic traits was administered in this study.

For this study, we used five low-stakes traffic scenarios. Two of them were the same as Study 1 (Pet and Intersection), while the remaining involved a cow crossing the street as a man drives his pregnant wife to the hospital (Cow), a female driver who must yield to an ambulance on a narrow mountain road (Ambulance), and a fire marshal managing a riot (Crowd). All the scenarios were developed using Unity (version 2020.3.18f1) and adapted for 360-degree projection. A total of 30 vignettes were created—six for each scenario (Pet, Intersection, Cow, Ambulance, and Crowd). Unlike Study 1, fully positive (A+D+C+) and fully negative (A–D–C–) vignettes were excluded from data collection.

Participants first viewed a training vignette to familiarize themselves with the procedure; responses to this vignette were excluded from the analysis. They then watched and responded to three vignettes from each of the five scenarios (15 in total). To measure participants' moral judgment, we adopted the same scale of personal and social acceptability as in Study 1.

#### 7.1.3 Analytic plan

Primary analyses were conducted in SAS 9.4 using linear mixed-effects models to account for repeated observations within participants. Because Study 2 collected separate ratings of personal moral judgment (MJP) and social moral judgment (MJS), we first evaluated whether these measures should be combined by fitting a mixed-effects model with rating type (MJP vs. MJS) as a fixed effect and participant as a random effect. The convergence of the two measures was additionally evaluated using the intraclass correlation coefficient (ICC). Based on this assessment, MJP and MJS were retained as separate dependent variables for subsequent analyses.

To test H1–H3, separate mixed-effects models were estimated for MJP and MJS. Agent (A), Deed (D), and Consequence (C) valence were entered as fixed effects, with a random intercept for participant to account for repeated observations. Least-square means were used to estimate differences between positive and negative component valences.

To examine whether individual moral preferences moderate moral judgments (R1–R3), mixed-effects models tested Framework, Vignette, and their interaction. Planned contrasts compared positive and negative component valences within their theoretically corresponding frameworks: agent within virtue ethics, Deed within deontology, and Consequence within consequentialism. These analyses were conducted separately for personal and social moral judgments.

A sensitivity power analysis was conducted in R version 4.5.2 using the *pwr* package (version 1.3-0) (Champely, 2020) to estimate the smallest detectable within-participant effect given the final sample (*n* = 146). Assuming α = 0.05 and 80% power, the design could detect an effect of approximately *d* = 0.23 (*f* = 0.12, η^2^_*p*_ = 0.01), corresponding to an effect of relatively modest magnitude.

As described in Study 1, supplementary Bayesian mixed-model comparisons were conducted for focal non-significant findings using the *BayesFactor* package (version 0.9.12-4.8) (Morey and Rouder, 2026). Models containing the focal effect were compared with otherwise matched reduced models excluding the effect, with participant treated as a random factor. BF_01_ represents the relative evidence for the reduced model, whereas BF_10_ represents evidence for the model containing the focal effect.

### 7.2 Results

#### 7.2.1 Main effects of Agent, Deed, and Consequence on moral judgment (H1–H3)

Personal and social moral-acceptability ratings differed significantly, *F* (1, 145) = 10.2, *p* = 0.002. Personal ratings (MJP) were higher overall (*M* = 5.69, *SE* = 0.17) than social ratings (MJS; *M* = 5.18, *SE* = 0.15). The intraclass correlation between rating types was moderate (*ICC* = 0.35, σ^2^_between_ = 2.43; σ^2^_residual_ = 4.42). Accordingly, MJP and MJS were analyzed separately. This differs from Study 1, in which the high correspondence between personal and social ratings supported their aggregation into a composite measure.

Agent valence strongly influenced both forms of moral judgment. Positively portrayed drivers (A+) were rated more morally acceptable for society (*M* = 6.58, *SE* = 0.10) than negatively portrayed drivers (A–; *M* = 3.75, *SE* = 0.10), *F* (1, 2004) = 352.0, *p* < 0.001, Δ*M* = 2.83, *d* = 1.16. The corresponding difference for personal moral judgment was also significant (A+: *M* = 7.08, *SE* = 0.10; A–: *M* = 3.81, *SE* = 0.10), *F* (1, 2010) = 467.6, *p* < 0.001, Δ*M* = 3.27, *d* = 1.16. These results support H1 for both MJS and MJP.

Consistent with H2, rule-following deeds (D+) received higher social acceptability ratings (*M* = 5.61, *SE* = 0.10) than rule-violating deeds (D–; *M* = 4.71, *SE* = 0.10), *F* (1, 2007) = 35.20, *p* < 0.001, Δ*M* = 0.90, *d* = 0.32. The same pattern occurred for personal acceptability (D+: *M* = 5.88, *SE* = 0.10; D–: *M* = 5.00, *SE* = 0.10), *F* (1, 2014) = 33.60, *p* < 0.001, Δ*M* = 0.88, *d* = 0.31.

Finally, positive consequences (C+) were rated more morally acceptable for society (*M* = 5.57, *SE* = 0.10) than negative consequences (C–; *M* = 4.75, *SE* = 0.10), *F* (1, 2004) = 29.90, *p* < 0.001, Δ*M* = 0.83, *d* = 0.29. Positive consequences likewise received higher personal acceptability ratings (*M* = 5.81, *SE* = 0.10) than negative consequences (*M* = 5.08, *SE* = 0.10), *F* (1, 2010) = 23.20, *p* < 0.001, Δ*M* = 0.73, *d* = 0.26. These results support H3 for both MJS and MJP.

#### 7.2.2 Moderation of Agent, Deed, and Consequences by moral preferences (R1–R3)

For personal moral judgment, significant main effects were observed for Vignette, *F* (5, 688) = 103.90, *p* < 0.001, and Framework, *F* (2, 138) = 6.22, *p* = 0.003. The Vignette × Framework interaction was not statistically significant, *F* (10, 688) = 1.73, *p* = 0.071. A supplementary Bayesian model comparison strongly favored the model without the interaction, BF_01_ = 62.57 (BF_10_ = 0.016). Thus, the analysis did not provide evidence that moral frameworks generally moderated responses across vignettes. Nevertheless, the prespecified planned contrasts revealed distinct within-framework patterns.

Among participants classified as virtue ethicists, A+ vignettes were rated more morally acceptable than A– vignettes, Δ*M* = 1.30, *SE* = 0.09, *t* (688) = 14.10, *p* < 0.001, *d* ≈ 0.46. Among participants classified as deontologists, D+ vignettes were rated lower than D– vignettes, Δ*M* = −0.39, *SE* = 0.09, *t* (688) = −4.28, *p* < 0.001, *d* ≈ 0.14, a pattern opposite the direction anticipated by R2. Among consequentialists, the C+ vs. C– contrast was small and non-significant, Δ*M* = −0.09, *SE* = 0.10, *t* (688) = −0.96, *p* = 0.340, *d* ≈ 0.03. A supplementary Bayesian mixed-model comparison modestly favored the model without a Consequence-valence effect, BF_01_ = 2.82 (BF_10_ = 0.355). Because the omnibus Vignette × Framework interaction was not significant, these planned contrasts should be interpreted as within-framework patterns rather than evidence of statistically significant moderation by moral framework.

A similar pattern emerged for social moral judgment. Participants with virtue-ethical preferences rated A+ vignettes 1.09 points higher than A– vignettes (*SE* = 0.09), *t* (688) = 11.70, *p* < 0.001, corresponding to a medium effect (β = 0.38). Among deontologists, D+ vignettes were rated 0.22 points lower than D– vignettes (*SE* = 0.09), *t* (688) = −2.37, *p* = 0.018 (β = −0.08), again opposite the direction predicted by R2. The C+ vs. C– contrast among consequentialists was also small and non-significant, Δ*M* = −0.10, *SE* = 0.10, *t* (688) = −1.06, *p* = 0.290 (β = −0.04). Here, the supplementary Bayesian comparison was inconclusive, BF01 = 1.21 (BF10 = 0.83), providing little evidence favoring either model.

### 7.3 Study 2 discussion

The primary goal of this study was to test the ADC model of traffic moral judgment using a different participant sample and a more immersive experimental setting. The results supported the model's core hypotheses (H1–H3). Overall, the Agent component showed the strongest effect on moral judgment, with moderate effects observed for Deed and Consequence.

Like in the previous study, we examined whether individual moral preferences influenced evaluations of the three components (R1–R3). The omnibus Vignette × Framework interaction was not significant, and the supplementary Bayesian analysis provided strong evidence favoring the model without the interaction. Thus, Study 2 did not provide evidence of general moderation by moral framework. However, the prespecified within-framework contrasts revealed several notable patterns. Participants classified as virtue ethicists distinguished strongly between positively and negatively characterized agents, whereas participants classified as deontologists rated rule-violating deeds more favorably than rule-following deeds, contrary to the theoretically expected direction. Consequentialists did not significantly distinguish between positive and negative consequences. These contrast results should therefore be interpreted as descriptive patterns within the theoretically corresponding framework groups rather than as evidence that framework significantly moderated the effects of the ADC components.

## 8 General discussion

### 8.1 Summary of the findings

Our experiments aimed to investigate moral judgment in ordinary realistic traffic simulations. More specifically, we wanted to replicate previous findings supporting the ADC model of moral judgment in this domain.

The results of these two studies provide partial support for the core principles of the ADC model (see Table 3 for an overview). Across both studies, participants evaluated drivers more positively when they were characterized as good rather than bad, and when outcomes were favorable (i.e., safe arrival) rather than unfavorable (i.e., traffic collisions). The effect of rule compliance was more mixed: it emerged clearly in the second study, where rule-following behavior was evaluated more positively than rule violations, but was not consistently observed in the first study. In Study 1, this inconsistency appeared to reflect differences between presentation modalities, with visual scenarios yielding more positive evaluations of rule compliance, while textual scenarios showed a weaker or reversed pattern.

**Table 3:** Summary of the findings across the two studies.

Hypothesis/question	Study 1 (S1)	Study 2 (S2)
H1: Positively portrayed drivers (A+) will have higher moral acceptability as compared to negatively portrayed drivers (A-)	Supported in the textual and A/V conditions, and with a combined sample	Supported for social and personal moral judgment
H2: Traffic actions respecting rules (D+) will have higher moral acceptability as compared to actions violating rules (D-).	Supported in the A/V condition; reversed in the textual condition; not supported in the combined sample	Supported for social and personal moral judgment
H3: Traffic actions resulting in no collision (C+) will have higher moral acceptability as compared to traffic actions resulting in a collision (C-).	Supported in the textual condition and the combined sample, but not in the visual condition	Supported for social and personal moral judgment
R1: Will preference for *virtue ethics* moderate the effect of drivers' character (A) on moral acceptability judgments?	No interaction effect	No overall interaction; significant planned A contrast within virtue-ethics group
R2: Will preference for *deontological ethics* moderate the effect of traffic rules (D) on moral acceptability judgments?	No interaction effect	No overall interaction; significant planned D contrast within deontology group, opposite predicted direction
R3: Will preference for *consequentialism* moderate the effect of traffic collision (C) on moral acceptability judgments?	No interaction effect	No overall interaction; planned C contrast non-significant
R4: Will the presentation mode (Textual vs. A/V) moderate the effect of drivers' character (A) on moral judgment?	Positive interaction	N/A
R5: Will the presentation mode (Textual vs. A/V) moderate the effect of traffic rules (D) on moral judgment?	Positive interaction	N/A
R6: Will the presentation mode (Textual vs. A/V) moderate the effect of traffic collision (C) on moral judgment?	Negative interaction	N/A

In a previous study (Pflanzer et al., 2025), using the same textual vignettes and a similar sample of academic philosophers, each moral component (Agent, Deed, and Consequences) had a significant effect on moral judgment. Considering this result, we did not expect that professional ethicists would judge textual descriptions of traffic violations more positively than descriptions of rule compliance. Although we do not have a compelling explanation for this phenomenon, we suggest that participants could have found certain minor road infractions (such as passing over a stop sign) as mere conventional violations, especially if contrasted with other details of the story, such as the driver's character and the consequences of the drive, which were strongly effective. However, further research is needed to support and refine this explanation.

Another goal of our studies was to examine how different modes of presentation influence moral judgment. While the components of the ADC model were robustly influential across stimulus types, presentation format significantly moderated their effects. More specifically, audiovisual presentations tended to amplify sensitivity to agent-related information and rule violations, whereas textual vignettes emphasized outcome-based considerations. These findings are somewhat surprising insofar as one would expect that car collisions are visually impactful on the eye of an observer. Nevertheless, we need to consider that both scenarios we employed began with quite striking scenes to characterize the protagonist agent, both displaying cruelty and a lack of care for a vulnerable other: an abusive parent with a lack of care for their child (Intersection scenario) and a violent animal hater with a lack of care for a stray dog (Pet scenario). Possibly, these evocative agent characterizations might have led participants to overlook the outcome of the traffic action. Additionally, given the low-stakes nature of our scenarios, the traffic incidents were not lethal. So, future research will have to investigate if these results are replicated in high-stakes situations.

Study 2, involving a more immersive VR setting, further corroborated the effectiveness of agent-related visual stimuli. Indeed, in this experimental condition, the Agent component had the strongest effect among the components. This suggests, once again, that participants were particularly impressed by depictions of drivers' moral character, though not completely neglecting traffic rules and consequences.

To examine whether individual moral preferences moderated the influence of traffic components on moral judgment, we measured participants' orientations toward virtue ethics, deontological ethics, and consequentialism. In Study 1, no significant moderating effects of moral framework were observed. In Study 2, the omnibus Vignette × Framework interaction was likewise non-significant, and the supplementary Bayesian analysis strongly favored the model without the interaction. Nevertheless, the prespecified within-framework contrasts revealed several notable patterns: virtue ethicists strongly distinguished between positively and negatively characterized agents, deontologists rated rule-violating deeds more favorably than rule-following deeds, contrary to the theoretically expected direction, and consequentialists did not significantly distinguish between positive and negative consequences. These findings suggest potentially meaningful within-framework response patterns but do not provide evidence that moral framework significantly moderated moral judgment. Future work with designs specifically powered to test cross-framework differences may help determine whether these within-framework patterns reflect reliable moderation effects.

### 8.2 Theoretical and practical implications

Our studies offer support for and refinement of the ADC model of traffic moral judgment, as recently theorized. However, the impact of each component is not uniformly robust, but instead appears to be context dependent. The one component that consistently influenced moral judgment across different samples and experimental designs was the driver's character. Encouragingly, our findings suggest that as the experimental environment becomes more ecologically valid, shifting from abstract textual vignettes to immersive visual simulations, results increasingly align with the core predictions of the ADC model. We recommend that future research continue to explore the conditions under which traffic rule violations and consequences meaningfully shape moral judgments, to further refine the model's applicability to real-world traffic contexts.

By comparing moral judgments across textual and visual versions of traffic scenarios, our study contributes to a deeper understanding of how the mode of presentation influences moral evaluation. Previous research using high-stakes sacrificial dilemmas, such as trolley problems, has shown that the type of stimulus—textual vignette versus immersive VR—can significantly shape moral judgments, with VR environments typically enhancing consequentialist responses (Patil et al., 2014; Faulhaber et al., 2019). In line with this body of work, we also found that the mode of presentation significantly moderated moral judgment in our low-stakes traffic scenarios. However, in contrast to earlier findings, our results suggest that when participants observed visual simulations of low-stakes traffic events, they placed greater emphasis on the character of the driver and adherence to traffic rules—moral dimensions more closely associated with virtue ethics and deontology rather than with consequentialism.

Although our studies did not directly examine moral judgments of AVs—as the scenarios involved human drivers—they non-etheless offer important insights for the ethics of driving automation, particularly in relation to designing AVs that align with common-sense moral expectations. As noted earlier, much of the existing literature has centered on the tension between consequentialist and deontological frameworks, often framed through the lens of the trolley problem (e.g., Awad et al., 2018). This paradigm has been instrumental in drawing public attention to AV ethics, sparking debate over whether AVs should be programmed to maximize overall utility or to follow universal moral principles. Our research contributes to this discussion in two important ways.

First, our findings highlight that, as in other moral domains, character-based considerations play a significant role in moral judgment of traffic scenarios—alongside deontological and consequentialist reasoning. This insight could be relevant for the design of AVs, as these vehicles may exhibit behavior perceived as reflecting traits such as aggressiveness or kindness on the road. To align AV decision-making with common-sense moral judgments, developers may need to incorporate proxies for these character-related attributes into AV programming, ensuring that vehicle behavior is not only functionally effective but also socially acceptable. As mentioned in Section 4, positive character cues could be incorporated into AV driving style through acceleration and braking modes, or courtesy in intersections. Of course, before becoming a direct recommendation for algorithm design, this finding needs to be confirmed in a first-person experimental setting. Our future research will investigate accordingly.

Second, by focusing on low-stakes traffic situations, our studies address an often-overlooked dimension of traffic moral judgment (Himmelreich, 2018; Borenstein et al., 2019; Cecchini et al., 2025). While dramatic trolley-like dilemmas tend to attract media attention and public debate, it is everyday traffic scenarios that AVs are far more likely to encounter. Investigating how people judge these routine situations advances the realism and practical relevance of AV ethics. Notably, our findings suggest that, regardless of individual moral preferences, participants tend to converge on core moral dimensions—namely, character, rule compliance, and outcomes—when evaluating traffic behavior. Whether such convergence persists in high-stakes scenarios and in a first-person setting remains an open question. Future research should directly compare low- and high-stakes contexts to explore how moral judgments may shift across different levels of urgency and risk.

### 8.3 Limitations and future lines of research

It is important to note that our study focuses on moral judgment rather than decision-making. We chose to examine third-person moral evaluations because these closely mirror how AVs will be assessed by external observers, such as other drivers, the public, and regulatory authorities, in real-world traffic contexts. Understanding how these third parties morally evaluate AV behavior is important for ensuring that AV decision-making aligns with societal expectations. Future progress in this area could be achieved by incorporating first-person methodologies, such as virtual driving simulators already used in previous research (Faulhaber et al., 2019; Sütfeld et al., 2019; Lucifora et al., 2020). In such setups, participants could adopt the role of drivers making morally relevant decisions, allowing researchers to examine whether their behavior aligns with the ADC model. Alternatively, participants could act as passengers in AVs programmed with ethical frameworks derived from the ADC model, while researchers measure their moral judgments and trust in the technology. These empirical approaches would help validate the ADC model within the traffic domain and support the shift from third-person moral judgment to first-person decision-making.

Another potential limitation concerns the strength of the agent manipulation used in several scenarios. To clearly operationalize the Agent component of the ADC model, the vignettes portrayed drivers with strongly positive or negative character cues. While this manipulation ensured that the agent variable was salient to participants, it may also have amplified the relative influence of Agent character compared to rule compliance (Deeds) or outcomes (Consequences). Future research could explore more subtle manipulations of driver character by, for example, varying driving style or situational intentions, to more precisely isolate the relative contributions of each component.

One relevant limitation of our studies lies in the composition of the participant samples, which were primarily drawn from WEIRD populations, i.e., Western, Educated, Industrialized, Rich, and Democratic societies (Henrich et al., 2010). While such samples are common in psychological and behavioral research, they raise important concerns about the generalizability of findings across cultures and social contexts. Moral judgment, particularly in domains such as traffic behavior and AV ethics, may vary significantly depending on cultural norms, legal expectations, and social trust in technology. Therefore, future research should strive to include more diverse and representative samples—both culturally and socioeconomically—to assess the robustness and universality of the ADC model.

Finally, our studies did not consider other relevant individual differences beyond moral preferences and psychopathic traits. For example, moral judgments (and the Deed component, particularly) could be influenced by driver's license status, daily driving frequency, and knowledge of local traffic regulations. We recommend that future studies collect these data to prevent confounding variables.

## 9 Conclusion

With the development of driving automation technologies, traffic moral judgment has garnered significant attention. Recent literature highlights a growing need to understand how different stakeholders evaluate road traffic decisions, particularly in mundane situations. This paper contributes to the field by presenting two studies involving ordinary audio-visual and text-based traffic simulations. Drawing on the ADC model of moral judgment, the scenarios contained variations concerning the driver's character, traffic rules, and outcomes. Each of these factors had some influence on the participants' moral judgment, with a significant predominance of the agent's character. Additionally, the presentation format significantly moderated the effects of the component: visual stimuli more effectively highlighted drivers' character and traffic rule violations, whereas textual vignettes more strongly conveyed the significance of outcomes in traffic situations.

Future research should examine whether the predominance of agent-related considerations persists in high-stakes scenarios by comparing moral judgments across situations that systematically vary agents, deeds, and consequences. A key direction for further research is the translation of the ADC model from a framework for moral judgment to one that can guide algorithmic decision-making in AVs.

## Data Availability

The raw data supporting the conclusions of this article will be made available by the authors, without undue reservation.

## Footnotes

1 Participants were excluded if their responses showed extremely low variability (standard deviation < 0.5) across the set of moral judgment ratings. This criterion was applied only to the vignette evaluation items and not to the PPIMT or demographic measures. Extremely low variance across multiple scenario ratings is commonly used as an indicator of non-differentiated responding or “satisficing” behavior in online surveys (Leiner, 2019). The threshold of 0.5 SD was selected as a conservative cutoff to identify participants who provided nearly identical responses across scenarios, suggesting limited engagement with the task.
