# Supplementary material for: Goodness behind a wheel: moral judgment in realistic traffic simulations featuring Agents, Deeds, and Consequences
Source: Front Psychol. 2026 Sep 28;17:1885843. doi: 10.3389/fpsyg.2026.1885843 (PMC13654839; doi:10.3389/fpsyg.2026.1885843)
Supplement: Supplementary file 1 [file Supplementary_file_1.docx]

# Appendix: Textual vignettes (Study 1)

**Intersection**

A caring mother who tries their best to help their children excel is driving their children to middle school during morning rush hour traffic (A+)/A negligent mother, frustrated with their child for missing the school bus due to oversleeping, hits the child but agrees to drive them to school (A-).
The mother is approaching a busy intersection when they see the traffic light ahead turn yellow. As the mother presses on the brakes to slow down, the car only reaches a complete stop after it has already crossed into the intersection, leaving the front end of the car sticking out towards oncoming traffic.
The parent then makes a split-second decision to reverse the car back behind the line so as not to be hit by approaching vehicles (D+)/The parent then makes a split-second decision to forcefully accelerate to try to pass through the intersection (D-).
In the end, all is well, and the children arrive at school with plenty of time to spare (C+)/the mother's car is rear ended and the children miss school (C-).

**Pet**

A young man offers to take his disabled mother’s sick cat to the local animal hospital. As he leaves his apartment he notices a dog sitting passively near his car. Approaching the dog, he sees that it is dirty and has no collar. Instead of ignoring the stray, he decides to take it along with him to the vet (A+)/A young man, angry with his disabled mother for insisting he take her sick cat to the local animal hospital, reluctantly agrees. As he leaves, he notices a stray dog sitting passively near his car. Before he gets into his car he takes his frustration out on the dog by kicking it until it runs away (A-).
A little while after leaving his apartment complex, he passes a flashing sign indicating that a stop sign is approaching. Although there are rarely drivers on the main road he is about to cross, he comes to a stop at the intersection. After being stopped for a few seconds, he is sure that no traffic is coming from either direction, and drives through the intersection. (D+)/Instead of slowing down, he chooses to speed up because he is familiar with this intersection and there is typically no one driving on the main road he is about to cross (D-).
He arrives at the hospital without issue where the cat is successfully treated with antibiotics (C+)/Seconds after he stops he hears the screeching of tires and is jerked forward as a reckless driver slams into him from behind. Neither driver suffers any major injuries, but both vehicles are rendered useless (C-).
